# MiR-99a-3p downregulates TRIM21 to promote gastric cancer development

**DOI:** 10.1007/s11010-024-05005-0

**Published:** 2024-05-08

**Authors:** Ling He, Jiaoli Zhou, Doukun Ding, Yongjing Jiang, Rui Yang, Zhiming Li

**Affiliations:** 1 Department of Gastroenterology, Affiliated Hospital of Jiangxi University of TCM, No.445, Bayi Road, Nanchang, 330006 Jiangxi China; 2 Jiangxi University of TCM, No.56, Yangming Road, Nanchang, 330006 Jiangxi China; 3 Department of Oncology, Affiliated Hospital of Jiangxi University of TCM, No.445, Bayi Road, Nanchang, 330006 Jiangxi China

**Keywords:** Gastric cancer, MiR-99a-3p, TRIM21

## Abstract

Gastric cancer (GC) stands as one of the most formidable malignancies worldwide. It is well-established that miRNAs play a crucial role in the initiation and progression of various human cancers. Among these, miR-99a-3p has been implicated in the pathogenesis of GC. In the context of our study, we embarked on the comprehensive examination of miR-99a-3p expression in GC cells. Additionally, we sought to establish a correlation between miR-99a-3p expression levels and the overall survival (OS) of GC patients, and our findings hinted at its potential role in predicting an unfavorable prognosis. To further investigate the functional implications of miR-99a-3p in GC, we conducted a series of cell-based experiments after successfully knocking down miR-99a-3p. These investigations uncovered a substantial inhibition of cellular events associated with tumor progression. Moreover, employing TargetScan, we identified Tripartite motif-containing protein 21 (TRIM21) as a putative target with a binding site for miR-99a-3p. Subsequent dual-luciferase reporter gene assay confirmed the direct interaction between miR-99a-3p and TRIM21. Western blot analysis validated the alteration in TRIM21 expression levels, revealing an upregulation upon miR-99a-3p knockdown. Building on these molecular findings, we extended our investigations to human GC tissues, where we observed a downregulation of TRIM21, which, notably, correlated with shorter overall survival. Lastly, to further solidify our conclusions, we conducted a series of in vitro and in vivo rescue experiments, collectively suggesting that miR-99a-3p promoted the progression of GC cells through the downregulation of TRIM21. In summary, our study comprehensively explored the role of miR-99a-3p in GC, revealing its association with unfavorable patient outcomes, functional implications in tumor progression, and a direct regulatory relationship with TRIM21. These findings collectively underscore the significance of miR-99a-3p in the pathogenesis of GC and present a potential therapeutic avenue for further investigation.

**Supplementary Information:**

The online version contains supplementary material available at 10.1007/s11010-024-05005-0.

## Introduction

Gastric cancer (GC) holds the unfortunate distinction of being the fifth most frequently diagnosed malignancy worldwide, with the fourth-highest mortality rate, thus underscoring its significance as a critical public health concern [1, 2]. In clinical practice, a combination of surgical intervention, radiotherapy, and chemotherapy represents the current therapeutic arsenal for the management of GC [3]. Despite significant advancements in these treatment modalities, the issue of recurrence persists, often leading to a bleak prognosis for afflicted individuals [4, 5]. This persistent clinical challenge necessitates a deeper understanding of the etiological factors contributing to GC and underscores the urgency in identifying novel therapeutic targets. Such insights are indispensable for the development of innovative treatment strategies that could potentially improve the overall outlook for GC patients.

MicroRNAs (miRNAs) have been reported to be frequently located in chromosomal regions associated with cancer susceptibility and genomic instability [6]. Dysregulation of these miRNAs, encompassing both tumor-suppressive and oncogenic types, plays a pivotal role in the initiation and progression of various malignancies [7]. One such miRNA of significance is miR-99a-3p, which is transcribed from the long arm of chromosome 21. It has been implicated in the advancement of malignant tumors and holds promise as a prognostic indicator. Notably, studies have demonstrated its relevance in different cancer types. In non-small cell lung cancer, miR-99a-3p upregulation, such as in the case of D261, has been shown to suppress cell growth and inhibit tumor development [8]. MiR-99a-3p targets integrin subunit alpha 2 (ITGA2), affecting metastasis in papillary thyroid carcinoma [9]. Its role in colorectal cancer is associated with overall patient survival [10]. MiR-99a-3p loaded exosomes have been found to influence cell proliferation, metastasis, and angiogenesis in invasive pituitary adenoma [11]. Research has also indicated its influence on prostate cancer progression by targeting non-SMC condensin I complex subunit G (NCAPG) [12]. However, despite these diverse associations in various cancer types, limited research has explored the specific effects and mechanisms of miR-99a-3p in GC.

In our investigation, we have identified a significant upregulation of miR-99a-3p in gastric cancer. Furthermore, our research has unveiled the multifaceted roles played by miR-99a-3p in the pathogenesis of gastric cancer. The overarching objective of this study is to delve into the underlying mechanisms that drive the development of gastric cancer and to pinpoint novel molecular targets that hold promise for the treatment of this condition.

## Materials and methods

### Ethics statement

This study received approval from the Ethical Committee of the Affiliated Hospital of Jiangxi University of Traditional Chinese Medicine (Approval No. 2020L010). Informed consent was obtained from all study participants. Furthermore, all animal experiments conducted in this study adhered to the guidelines outlined in the European Parliament Directive (2010/63/EU).

### Database and online tools

We examined the association between miR-99a-3p and TRIM21 and the overall survival (OS) of patients with gastric cancer. To categorize high and low expression levels of miR-99a-3p and TRIM21, we employed median based on data sourced from the Starbase website (https://rnasysu.com/encori/panCancer.php). Furthermore, we used TargetScan (http://www.targetscan.org/vert_72/) to predict the potential binding site of miR-99a-3p and TRIM21.

### Cell lines and animals

In this study, human normal gastric mucosal cell GES1, and gastric cancer cells AGS and MGC-803 were utilized. These cell lines were procured from the American Type Culture Collection (ATCC) in December 2020. GES1 and AGS cells were cultured in 1640 medium supplemented with 10% fetal bovine serum (FBS). MGC-803 cells were cultivated in RPMI-1640 medium, supplemented with 10% FBS and 1% double antibody.

Six-week-old female BALB-c nude mice, weighing approximately 21 g, were sourced from Jiangsu Jicui Yaokang Biotechnology Co., Ltd. (Jiangsu, China). The mice were housed in cages (5 mice per cage) and maintained at a temperature of 22–25 °C, with a humidity level of 50–60%, and a 12-h light/dark cycle. Adequate water and food were provided to ensure their well-being.

### Immunohistochemistry (IHC)

A gastric cancer tissue microarray was sourced from Shanghai Outdo Biotech Co., Ltd (Shanghai, China). The samples underwent deparaffinization, followed by repair with 1 × EDTA (Beyotime Biotechnology Co., Ltd, Shanghai, China), and were subsequently blocked with 3% H_2_O_2_ for 5 min. Subsequently, the sections were incubated with primary and secondary antibodies overnight at 4 °C. DAB (3,3'-diaminobenzidine) staining was applied for 5 min, and counterstaining was performed with hematoxylin (Baso Diagnostics Inc., Zhuhai, China) for 10–15 s. The extent and intensity of staining were assessed as previously outlined [13]. For quantitative analysis, an IHC score, independently determined by three pathologists, was employed. High and moderate expression parameters were defined in relation to the median IHC scores of all tissues. Detailed information about the antibodies used is provided in Table S1.

### Target design of shRNA and the construction of lentivirus vector for target gene

To construct a lentiviral vector targeting miR-99a-3p (shmiR-99a-3p with the sequence CAGACCCATAGAAGCGAGCTTG) and a shRNA vector against TRIM21 (shTRIM21 with the sequence TGGCTCCCTCATCTACTCCTT), a specific design was employed. For the negative control (NC), a scramble sequence (TTCTCCGAACGTGTCACGT) was used. Prior to infection, cells were seeded in 6-well plates and allowed to grow for 24 h. Lentivirus at a concentration of 1 × 10^8^ TU/mL was added to infect 2 × 10^5^ cells under ENI.S + Polybrene conditions for 72 h. The lentiviral vector included a Puromycin resistance gene, which was used for the selection of cell populations after lentiviral infection. Furthermore, the lentiviral construct featured a GFP marker, which facilitated the observation of cell infection efficiency. In co-silencing experiments, both shTRIM21 and shmiR-199a-3p were transfected simultaneously, and the cells were incubated for 24 h post-transfection. Subsequently, a 5-day purine selection process was implemented to ensure the successful co-silencing of the target genes.

### Quantitative real-time PCR (qRT-PCR)

Total RNA was isolated using TRIzol reagent (Sigma, St. Louis, MO, USA) according to the manufacturer's instructions. Reverse transcription was conducted using the Promega M-MLV Kit (Promega Corporation, Madison, Wisconsin, USA). The PCR system comprised 10 μL and was performed with SYBR Green qPCR Master Mix (Vazyme, Nanjing, Jiangsu, China). Relative expression levels were calculated using the 2^−△△Ct^ method, with U6 and GAPDH serving as internal controls. The primers sequences used in qPCR were as follows (5’–3’): The forward primer of miR-99a-3p was 5′–CAAGCTCGCTTCTATG–3′, the reverse primer of miR-99a-3p was 5′–GTGCAGGGTCCGAGGT–3′; the forward primer of U6 was 5′–CTCGCTTCGGCAGCACA–3′, the reverse primer of U6 was 5′–CTCGCTTCGGCAGCACA–3′. The forward primer of TRIM21 was 5′–TCAGAGCTAGATCGAAGGTGC–3′, the reverse primer of TRIM21 was 5′–ACTCACTCCTTTCCAGGACAAT–3′; the forward primer of GAPDH was 5′–TGACTTCAACAGCGACACCCA–3′, the reverse primer of GAPDH was 5′–CACCCTGTTGCTGTAGCCAAA–3′.

### Western blot

The cells underwent PBS washing and subsequent lysis with 1 × Lysis Buffer (Cell Signal Technology, Danvers, MA). The protein extraction from murine tumors involved washing the tissue blocks with cold PBS to remove blood contaminants, followed by cutting them into small pieces and placing them in grinding tubes with grinding beads. The tissue lysis buffer supplemented with protease inhibitors was added, and the samples were homogenized using a low-temperature high-speed tissue homogenizer. After grinding, samples were kept on ice for 30 min for lysis, with occasional agitation or sonication. Subsequently, the lysates were centrifuged at 4 °C, 12,000 g for 10 min, and the supernatant containing total protein was collected for quantification using the BCA protein assay kit. The resulting samples were separated by 10% SDS-PAGE and transferred onto PVDF membranes. Following this, the membranes were blocked with 5% skim milk in Tris-Buffered Saline Tween (TBST) buffer at room temperature for 1 h. Subsequently, the membranes were incubated with primary antibodies and then secondary antibodies at room temperature for 2 h. Finally, the membranes were visualized using the ECL + plusTM Western blotting system kit. Detailed information about the antibodies used can be found in Table S1.

### Dual-luciferase reporter assay

The online TargetScan was utilized for predicting the binding sites of miR-99a-3p within the 3' untranslated regions (UTR) of TRIM21 mRNA (TRIM21-wild-type or TRIM21-wt). A mutated version (CCGGATtttgGCAAGtagatccT) of TRIM21 was generated, introducing alterations to the TRIM21 3' UTR, resulting in the TRIM21-mutant (TRIM21-mut). The TRIM21-wt/mut plasmids were then integrated into the 3'-end of the pGL3-basic luciferase vector. Subsequently, the TRIM21-wt/mut-pGL3 plasmids and the pRL-TK control were co-transfected into 293 T cells, followed by transfection with either 1 pmol miR-99a-3p mimics or NC mimics. After 48 h post-transfection, luciferase activity was assessed using the Dual Luciferase Reporter Assay System (Promega, Madison, WI, USA) in accordance with the manufacturer’s protocol. 75 μl of Dual-Glo® Reagent was added to the plate and incubated at room temperature for 10 min to initiate the reaction with Firefly luciferase, enabling the measurement and recording of Firefly luciferase activity, which serves as the reporter gene luminescence value. Following this, 75 μl of Stop & Glo® Reagent was added to the wells and allowed to incubate at room temperature for another 10 min. The Renilla luciferase activity, serving as the internal control value, was then measured and recorded. The obtained data were expressed as relative luciferase activity (Firefly luciferase activity/Renilla luciferase activity).

### RNA immunoprecipitation (RIP) assay

The effective pulldown of miR-99a-3p by AGO2 has been demonstrated in a previous report [12]. RIP experiments were performed using the Magna RIP™ RNA-Binding Protein Immunoprecipitation Kit (Millipore, USA). Initially, AGS cell lysates were prepared by incubating cells in RIP lysis buffer (supplemented with RNase and protease inhibitors) to ensure the integrity of RNA–protein complexes. The lysates were then subjected to immunoprecipitation (IP) using either 6 µg of anti-AGO2 antibody (specific to Argonaute 2 protein) or normal anti-IgG as a negative control. The IP reaction mixture was gently rotated overnight at 4 °C to facilitate antibody binding to the target RNA–protein complexes. Following this, RNA–protein complexes were subjected to preclearance with protein G Dynabeads, which selectively immobilize antibody-bound complexes. The Dynabeads were pre-washed to remove unbound proteins and contaminants before being added to the IP reaction mixture. After a brief incubation period, the Dynabeads-bound complexes were magnetically separated from the solution, and the supernatant was carefully removed. Next, the isolated RNA from the immunoprecipitated complexes was extracted using TRIzol reagent. The RNA was then treated with DNase to remove any contaminating genomic DNA and purified using an RNA purification kit. Subsequently, the purified RNA was subjected to reverse transcription to generate complementary DNA (cDNA) using a reverse transcription kit. The cDNA was utilized as a template for quantitative real-time polymerase chain reaction (qRT-PCR) analysis employing primers specific to TRIM21, the target of interest. The relative abundance of TRIM21 RNA associated with AGO2-bound complexes was determined by normalizing the qRT-PCR data to input RNA levels and compared to the IgG control to ascertain specific enrichment. The information about the antibodies is detailed in Table S1.

### Celigo cell counting assay

In the Celigo cell counting assay, cells were inoculated after infection at a density of 2000 cells per well into 96-well plates. Subsequently, the cells were cultivated for a consecutive 5-day period and assessed using the Celigo Meter from Nexcelom.

### CCK8 assay

For the CCK-8 assay, MGC-803 cells infected with specific lentiviruses were seeded into 96-well plates and allowed to incubate. After the designated incubation period, CCK-8 reagent was added to each well, followed by further incubation. Absorbance measurements at 450 nm were then taken to assess cell viability and proliferation. Higher absorbance values were indicative of increased cell proliferation.

### Wound-healing assay

AGS or MGC-803 cells were seeded in 6-well plates at a density of 100 cells per well following infection. Subsequently, the cells were cultured in a low-concentration serum medium. To induce a controlled wound, a 96-Well Wounding Replicator from VP Scientific was employed, which gently created a scratch by nudging upwards from the central area of the plate. Finally, the migration area of the cells was quantitatively assessed using Cellomics technology from Thermo.

### Transwell invasion assay

For the Transwell invasion assay, AGS or MGC-803 cells expressing specific lentiviruses were suspended in serum-free medium and introduced into the upper chamber of a Transwell insert featuring a Matrigel-coated membrane. The lower chamber was filled with medium containing chemoattractants. Following a designated incubation period, non-invading cells residing on the upper surface of the membrane were meticulously removed, while the invading cells present on the lower surface were fixed, stained, and subsequently enumerated.

### Flow cytometry assay

AGS or MGC-803 cells were seeded in 6-well plates at a volume of 2 mL per well and cultured for a duration of 5 days. Subsequently, 10 μL of Annexin V-APC was introduced in a light-protected environment and incubated for 10–15 min to facilitate staining. Finally, the extent of cell apoptosis was quantified using FACSCalibur (BD Biosciences, San Jose, CA, USA).

### Mouse tumor cell xenograft assay

The mice were randomly assigned to their respective experimental groups (*n* = 7/group), and 1 × 10^7^ AGS cells, carrying the specified lentiviruses, were subcutaneously injected into nude mice. Tumor volume was assessed using the formula: volume = π/6 × *L* × *W*^2^, where *L* represents the long diameter, and *W* represents the short diameter. After a 26-day period, the mice were euthanized to collect tumors for both weight determination and photographic documentation.

### Statistical analysis

All data were expressed as mean ± standard deviation (SD). Statistical analyses were conducted using GraphPad Prism 6.0 and SPSS 20.0. Student’s *t*-test was employed to compare data between two groups, while one-way ANOVA was utilized for comparisons involving three groups. Each experiment was repeated at least three times to ensure accuracy and reliability. A significance level of *P* < 0.05 was considered statistically significant.

## Results

### MiR-99a-3p was overexpressed in gastric cancer with poor prognosis

**Fig. 1:**
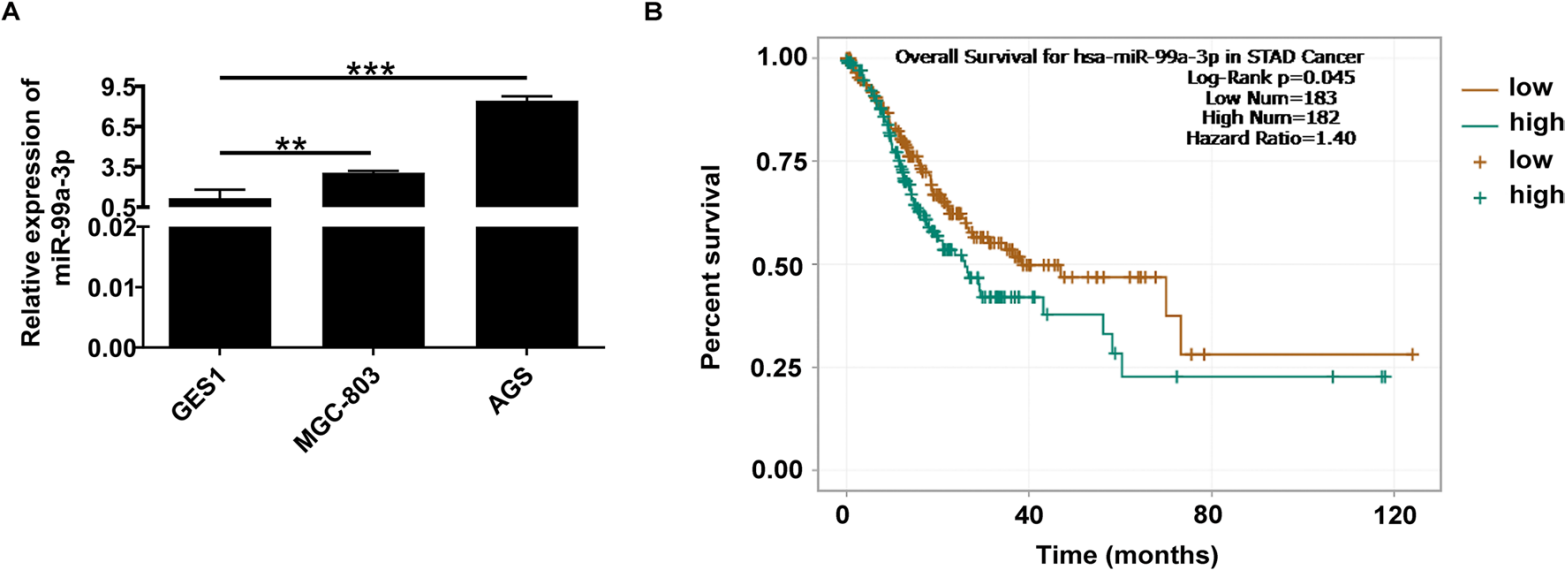
MiR-99a-3p was overexpressed in gastric cancer with poor prognosis. **A** The expression of miR-99a-3p in GES1, AGS as well as MGC-803 cell lines were tested via qRT-PCR assay. **B** The relationship between miR-99a-3p expression and GC patients’ prognosis was determined. High and low expression levels of miR-99a-3p were determined according to the median, data from the Starbase website. **P < 0.01, ***P < 0.001

We conducted qRT-PCR to quantify miR-99a-3p expression in GC cell lines. Our findings revealed that miR-99a-3p exhibited robust expression in AGS and MGC-803 cell lines in comparison to the normal gastric epithelial cells, GES1 (Fig. 1A). Additionally, a bioinformatics analysis was performed to assess the relationship between miR-99a-3p expression and patients' prognosis. Notably, the plotting of a Kaplan–Meier curve demonstrated that patients with elevated miR-99a-3p levels tended to have a shorter overall survival (OS) compared to those with lower miR-99a-3p expression. This suggests that miR-99a-3p might serve as a potential prognostic marker (*P* < 0.05, Fig. 1B). Taken together, these findings indicate an upregulation of miR-99a-3p in GC, potentially associated with a poor prognosis.

### Silencing miR-99a-3p affects cell events associated with tumor progression

**Fig. 2:**
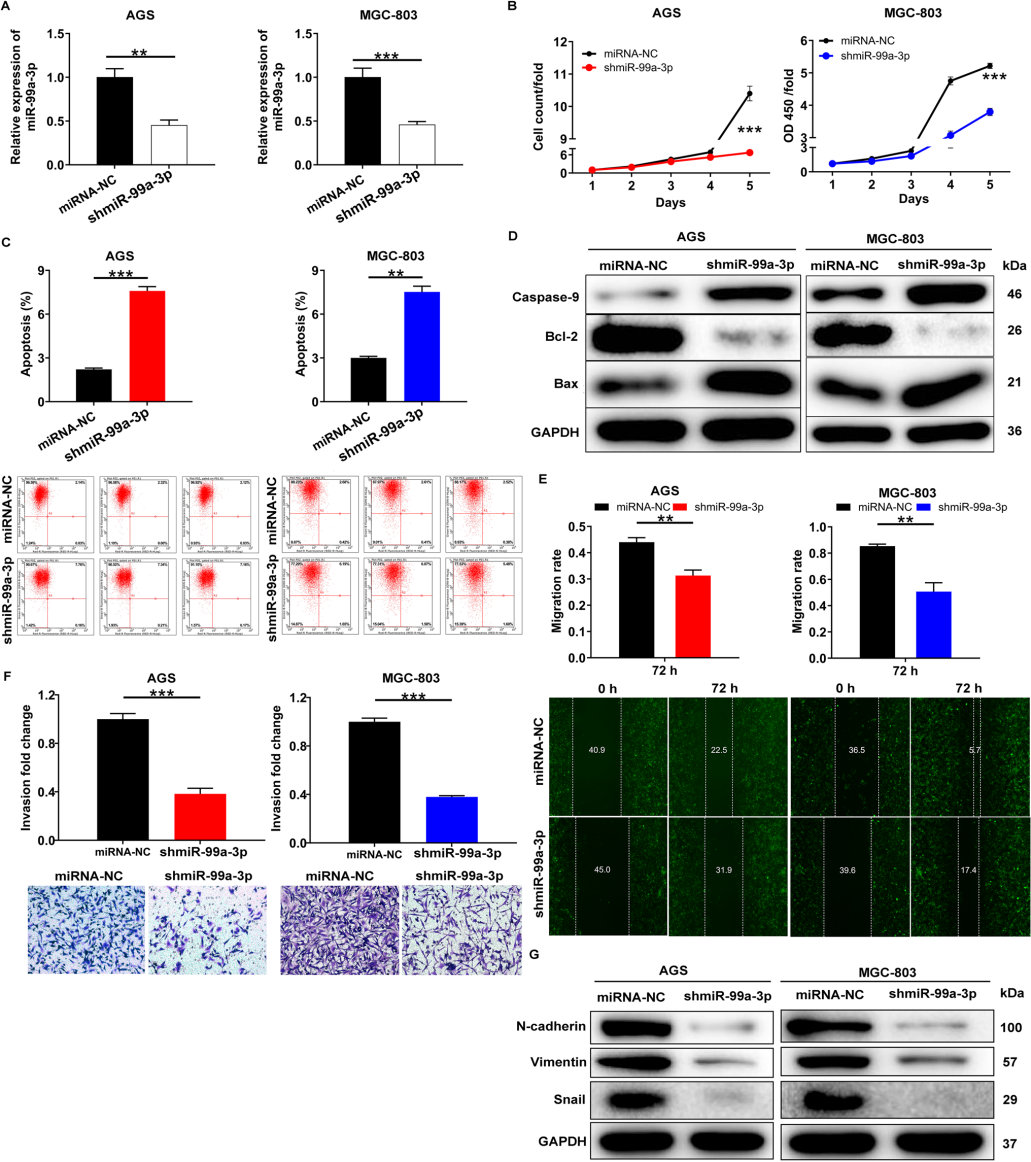
Silencing miR-99a-3p affects cell events associated with tumor progression. **A** qRT-PCR confirmation of miR-99a-3p downregulation in AGS and MGC-803 cell lines following miR-99a-3p inhibition. **B** Impact of miR-99a-3p knockdown on the proliferation of AGS and MGC-803 cells. Celigo counting assay for AGS cells and CCK8 assay for MGC-803 cells. **C** Assessment of cell apoptosis following miR-99a-3p depletion. **D** Western blot analysis of caspase-9, Bcl-2, and Bax protein levels in AGS and MGC-803 cells after miR-99a-3p inhibition. **E** Wound-healing assay demonstrating the suppression of cell migration as a result of miR-99a-3p downregulation. **F** Transwell invasion assay illustrating the reduced invasive capabilities of AGS and MGC-803 cells upon miR-99a-3p inhibition. **G** Western blot analysis of N-cadherin, Vimentin, and Snail expression in AGS and MGC-803 cells with miR-99a-3p downregulation. **P < 0.01, ***P < 0.001

To elucidate the roles of miR-99a-3p in GC, we first successfully suppressed miR-99a-3p expression in AGS and MGC-803 cell lines, as confirmed by qRT-PCR (Fig. 2A). Subsequently, we observed a substantial reduction in the proliferation of AGS and MGC-803 cells upon miR-99a-3p knockdown (*P* < 0.001, Fig. 2B). Moving on, we explored the impacts of miR-99a-3p on cell apoptosis. Our results revealed that depleting miR-99a-3p resulted in an increased number of apoptotic cells (Fig. 2C). To enhance the reliability of our apoptosis assessments, we conducted western blot analysis to quantify the expression levels of apoptosis-related proteins, such as caspase-9, Bcl-2, and Bax, in AGS and MGC-803 cells. Our findings demonstrated that the downregulation of miR-99a-3p led to an upregulation of caspase-9 and Bax, while concurrently causing a downregulation of Bcl-2 when compared to the control group (miRNA-NC) (Fig. 2D). Furthermore, we performed a wound-healing assay, which clearly indicated that miR-99a-3p downregulation resulted in the suppression of cell migration (Fig. 2E). Subsequently, we conducted transwell invasion assays to assess the invasive capabilities of AGS and MGC-803 cells following miR-99a-3p knockdown. The results demonstrated that the inhibition of miR-99a-3p led to a marked reduction in the invasion of AGS and MGC-803 cells when compared to the control group (miRNA-NC) (Fig. 2F). Additionally, these effects on cell migration and invasion were associated with decreased expression of N-cadherin, Vimentin, and Snail in both AGS and MGC-803 cell lines (Fig. 2G). In summary, the downregulation of miR-99a-3p in AGS and MGC-803 cell lines significantly inhibited cell proliferation, promoted apoptosis, suppressed cell migration, and reduced invasive capabilities, accompanied by alterations in the expression of key proteins associated with these processes.

### MiR-99a-3p targets TRIM21 in gastric cancer

**Fig. 3:**
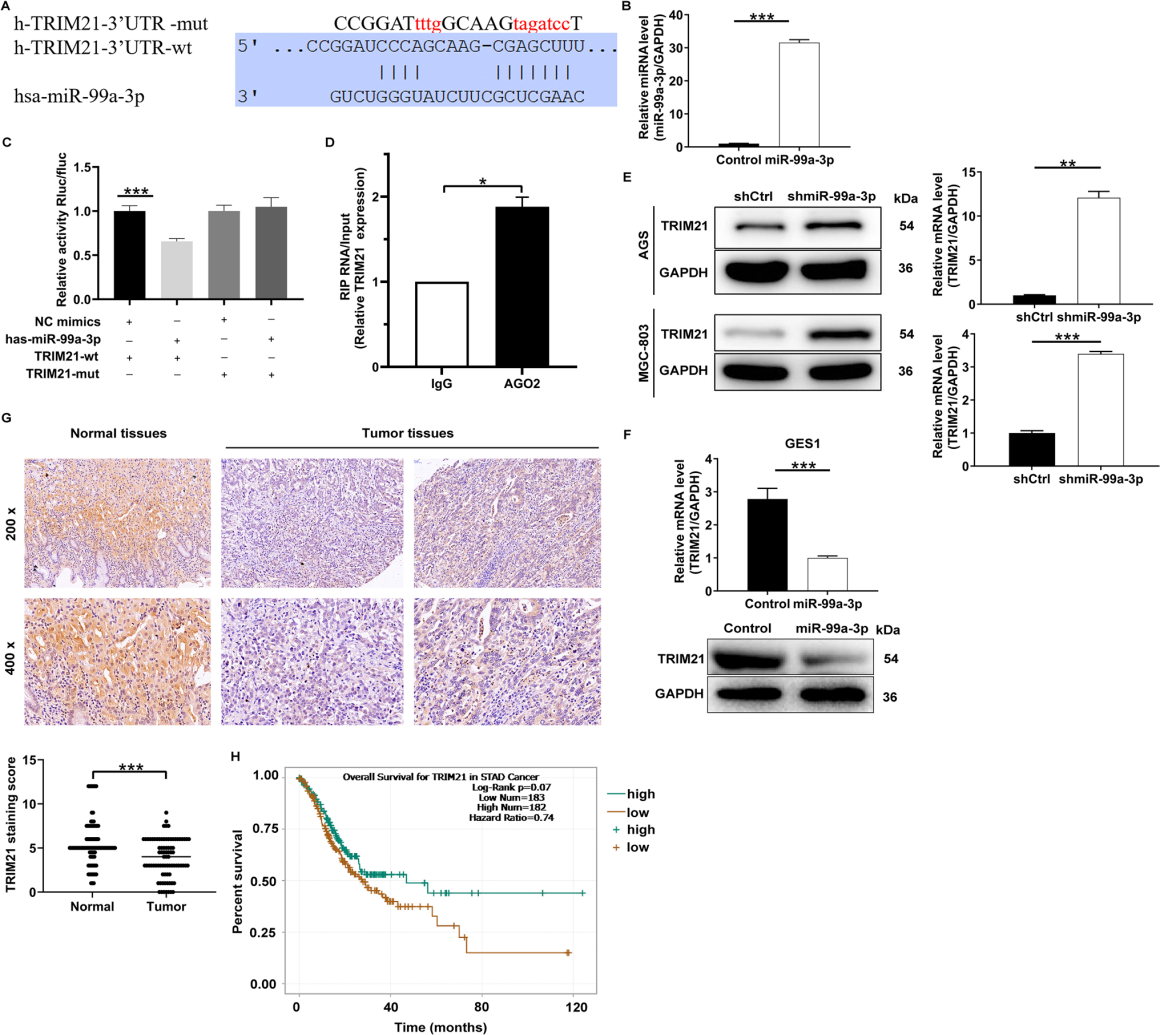
MiR-99a-3p targets TRIM21 in gastric cancer. **A** TargetScan analysis indicating the putative binding site of miR-99a-3p in the TRIM21 gene **B** Confirmation of miR-99a-3p overexpression through vector transfection. **C** Dual-luciferase reporter assay demonstrating the suppressive effect of miR-99a-3p overexpression on TRIM21 luciferase activity, with no effect observed when the binding site was mutated. **D** TRIM21 was retrieved by the AGO2 antibody in AGS cells. **E** Expression levels of TRIM21 at both the mRNA and protein levels following the suppression of miR-99a-3p in AGS and MGC-803 cells. **F** Effects of miR-99a-3p overexpression on TRIM21 levels in GES1 cells. **G** Immunohistochemical (IHC) staining demonstrating lower TRIM21 expression in tumorous gastric tissues compared to normal tissues. **H** Survival analysis indicating that patients with low expression of TRIM21 were more likely to experience a shorter overall survival. *P < 0.05, **P < 0.01, ***P < 0.001

In this section, we aimed to elucidate the molecular mechanisms underlying the pro-cancer activity of miR-99a-3p in GC. To this end, we employed TargetScan (http://www.targetscan.org/vert_72/) to screen for potential target genes of miR-99a-3p, revealing that TRIM21 harbored a binding site for miR-99a-3p (Fig. 3A). Subsequently, we conducted overexpression of miR-99a-3p using miR-99a-3p mimics (Fig. 3B). Furthermore, a dual-luciferase reporter assay demonstrated that the overexpression of miR-99a-3p suppressed the luciferase activity of TRIM21, an effect not observed when the binding site was mutated (Fig. 3C). We then performed RNA immunoprecipitation using AGO2 antibody to pulldown miR-99a-3p in AGS cell lysates. Subsequent qRT-PCR analysis confirmed the enrichment of TRIM21 in AGO2 immunoprecipitates (Fig. 3D). Upon the suppression of miR-99a-3p expression in AGS and MGC-803 cells, the level of TRIM21 was significantly elevated at both the mRNA and protein levels (Fig. 3E). We also conducted experiments to overexpress miR-99a-3p in GES1 cells. The data revealed that the overexpression of miR-99a-3p led to a downregulation of TRIM21 at both the mRNA and protein levels compared to the Control group (Fig. 3F). This provides valuable insights into the underlying mechanisms of miR-199a-3p's interaction with TRIM21. Furthermore, TRIM21 expression, as evidenced by IHC staining, was lower in tumorous gastric tissues compared to normal tissues (Fig. 3G). Additionally, patients with low expression of TRIM21 were more likely to experience a shorter overall survival (Fig. 3H). In conclusion, TRIM21 should be considered a target gene of miR-99a-3p in GC.

### MiR-99a-3p promotes gastric cancer by targeting TRIM21

**Fig. 4:**
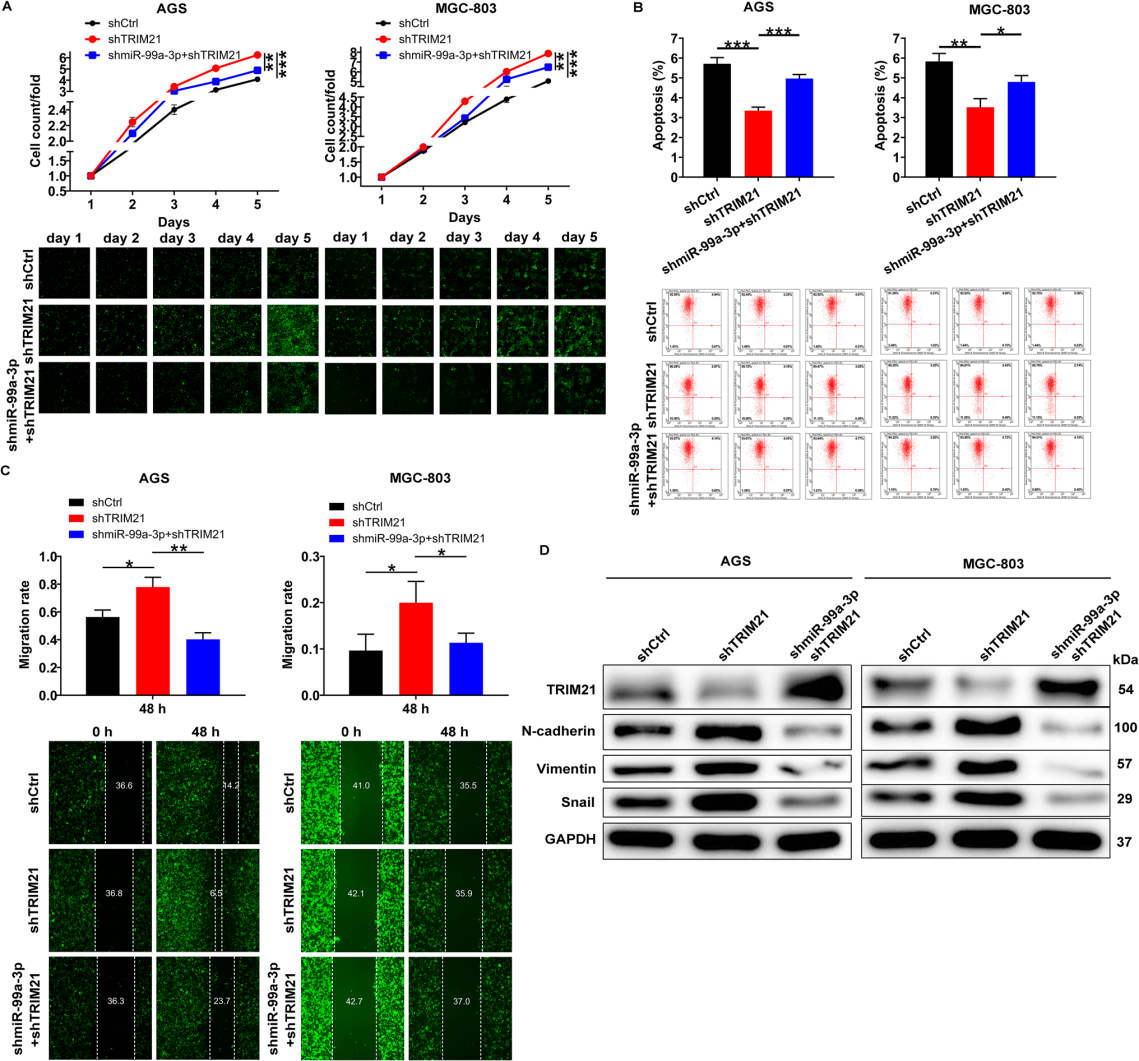
MiR-99a-3p promotes gastric cancer by targeting TRIM21 in vitro. **A** Following a 5-day co-transfection period, the impacts of TRIM21 and miR-99a-3p downregulation on the proliferation of AGS and MGC-803 cells were analyzed using Celigo counting assay. **B** After a 5-day co-transfection period, the impact of TRIM21 depletion on the apoptosis of AGS and MGC-803 cells, in conjunction with miR-99a-3p downregulation, was evaluated. **C** Following a 5-day co-transfection period, migration capability was evaluated in TRIM21-depleted cells subsequent to miR-99a-3p suppression. **D** After a 5-day co-transfection period, western blot analysis displaying the protein levels of N-cadherin, Vimentin, and Snail in AGS and MGC-803 cells between the shCtrl, shTRIM21, and shTRIM21 + shmiR-99a-3p groups. shCtrl: control; shTRIM21: TRIM21 downregulation; shmiR-99a-3p + shTRIM21: miR-99a-3p and TRIM21 downregulation. *P < 0.05, **P < 0.01, ***P < 0.001

After establishing the interaction between miR-99a-3p and TRIM21, we sought to investigate the impact of the miR-99a-3p/TRIM21 axis on the progression of GC. To do this, we generated AGS and MGC-803 cells using lentiviruses with specific constructs (shCtrl, shTRIM21 + shmiR-99a-3p, shTRIM21). When TRIM21 was downregulated, we observed an enhancement in the proliferation and migration of AGS and MGC-803 cells. Notably, this enhancement was subsequently reversed when we knocked down miR-99a-3p. Conversely, we observed that apoptosis was suppressed in TRIM21-depleted cells, and this effect was partially mitigated after silencing miR-99a-3p (Fig. 4A–C). Furthermore, we conducted a western blot analysis to evaluate the protein levels of TRIM21, N-cadherin, Vimentin, and Snail in AGS and MGC-803 cells. As expected, in comparison to the shCtrl group, the shTRIM21 group displayed a reduction in TRIM21 expression and an increase in the levels of N-cadherin, Vimentin, and Snail. Moreover, when comparing the shTRIM21 group to the shTRIM21 + shmiR-99a-3p group, we observed an elevation in TRIM21 expression and a concurrent reduction in the levels of N-cadherin, Vimentin, and Snail (Fig. 4D). These findings further supported the role of miR-99a-3p in modulating the expression of these proteins in the context of TRIM21 suppression. In summary, our results indicated that the miR-99a-3p/TRIM21 axis played a significant role in GC progression, affecting cellular proliferation, migration, and apoptosis, as well as the expression of critical proteins associated with the epithelial-mesenchymal transition (EMT) process.

**Fig. 5:**
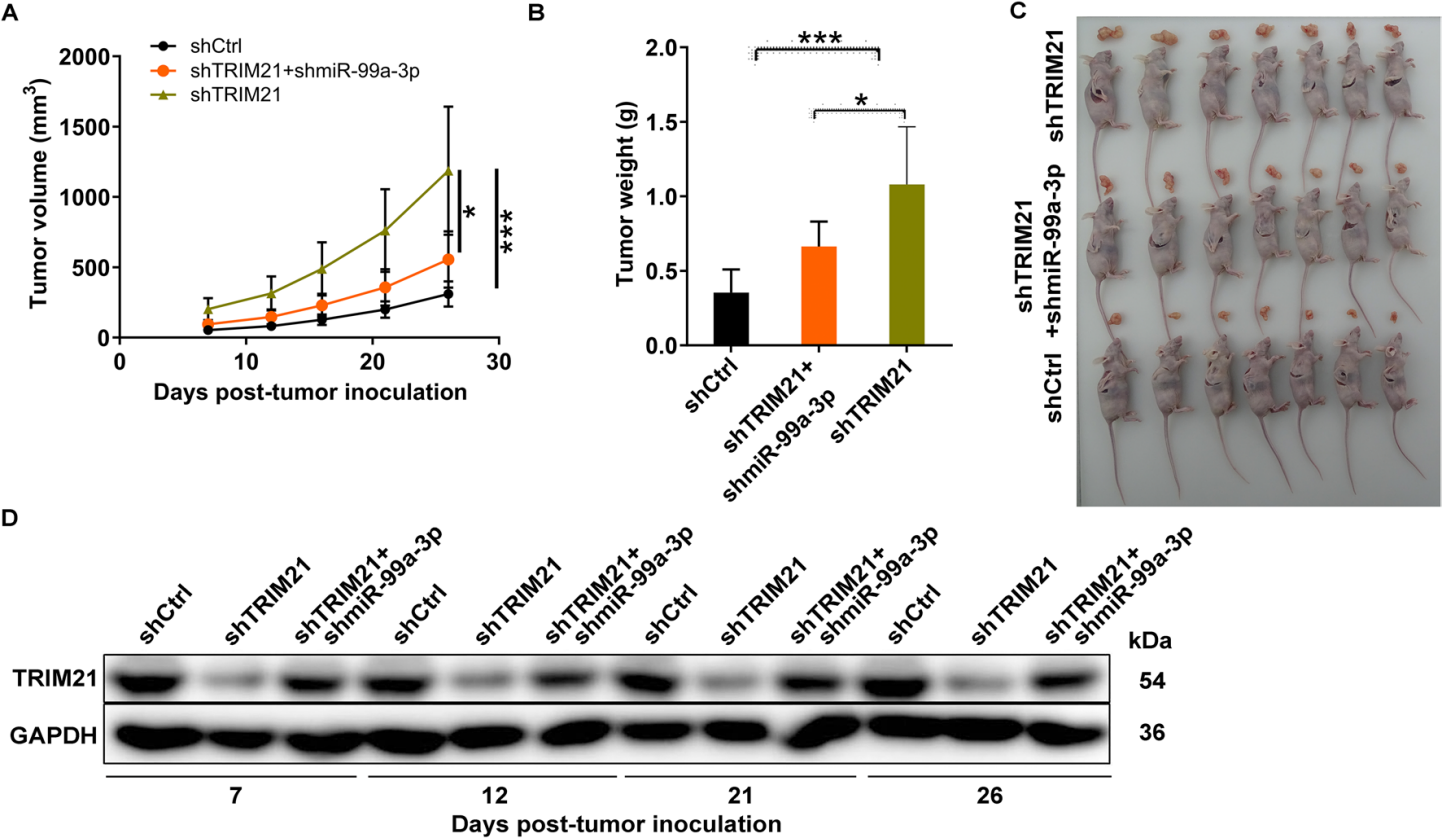
MiR-99a-3p promotes gastric cancer by targeting TRIM21 in vivo. **A**, **B** Tumor growth analysis in nude mice, illustrating the impacts of TRIM21/miR-99a-3p downregulation on tumor volume (**A**) and weight (**B**). **C** The photos of xenografts derived from MGC-803 cells were collected in the shCtrl, shTRIM21, and shTRIM21 + shmiR-99a-3p groups. **D** Western blot analysis of intratumoral TRIM21 protein levels in the shCtrl, shTRIM21, and shTRIM21 + shmiR-99a-3p groups. shCtrl: control; shTRIM21: TRIM21 downregulation; shmiR-99a-3p + shTRIM21: miR-99a-3p and TRIM21 downregulation. *P < 0.05, ***P < 0.001

In order to further investigate the tumorigenic potential of the miR-99a-3p/TRIM21 axis, we conducted subcutaneous injections of MGC-803 cells carrying the lentiviral constructs described earlier into the axilla of the right forelimb of nude mice. The results, as depicted in Fig. 5A–C, revealed that the downregulation of TRIM21 was associated with enhanced tumor growth in nude mice. Specifically, the mean tumor volume and weight of the MGC-803-shTRIM21 group were significantly increased when compared to the MGC-803-shCtrl group (*P* < 0.001). However, when simultaneously suppressing TRIM21 and miR-99a-3p, a marked reduction in tumor size of MGC-803-shTRIM21 xenografts was observed compared to the MGC-803- shTRIM21 group (*P* < 0.05). Subsequently, we performed western blot analysis to assess the levels of intratumoral TRIM21 protein in the shCtrl, shTRIM21, and shTRIM21 + shmiR-99a-3p groups. As expected, when compared to the control group, the shTRIM21 group displayed a reduction in TRIM21 protein levels. Notably, in comparison to the shTRIM21 group, the shTRIM21 + shmiR-99a-3p group exhibited an increase in TRIM21 protein levels (Fig. 5D). In conclusion, this study provides clear confirmation that miR-99a-3p plays a prominent role in the progression of GC by targeting TRIM21.

## Discussion

GC remains a significant global health challenge [14]. Clinical management typically involves a multifaceted approach, including surgery, radiotherapy, chemotherapy, and biological therapy [15]. Nevertheless, patients often confront a bleak prognosis, primarily due to the challenges associated with early diagnosis and the propensity for metastatic spread [16]. Hence, the development of innovative therapies for GC holds the potential to make a profound impact on a global scale.

According to previous studies, miR-99a-3p has exhibited tumor-suppressive effects in various human cancers. However, in contrast to these findings, our research identified an overexpression of miR-99a-3p in GC cells. This elevated expression was associated with reduced overall survival among GC patients. Upon silencing miR-99a-3p in GC cells, a significant suppression of cell proliferation and migration was observed in both AGS and MGC-803 cells, accompanied by an enhancement in apoptosis. Our findings led us to speculate that the role of miR-99a-3p in the pathogenesis of GC might differ from its role in other malignancies.

Our investigation into the target genes of miR-99a-3p revealed that TRIM21 contains a binding site for miR-99a-3p. Subsequently, through a dual-luciferase reporter gene assay, we confirmed that overexpression of miR-99a-3p could inhibit the luciferase activity of TRIM21 in the TRIM21-wt group but not in the TRIM21-mutant group. This suggests a binding relationship between TRIM21 and miR-99a-3p.As a member of TRIM family, TRIM21 locates on chromosome 11 [17] and encodes a ubiquitously expressed protein called RING-dependent E3 ligase [18]. TRIM21 has been widely discussed in cancer progression in many studies [19]. For example, Du et al*.* demonstrated that TRIM21, as the ubiquitin E3 ligase for Oct-1, could promote octamer-binding transcription factor 1 (Oct-1) ubiquitination and, consequently, reducing Oct-1 stability; in this way, a positive role played by TRIM21 in deactivating cancer stem cells and reducing treatment resistance [20]. In addition, TRIM21 was able to inhibit the expression of malignant transcription factors Sal-like family in breast cancer [21, 22]. Furthermore, TRIM21 level was low in diffuse large B-cell lymphoma and hepatocellular carcinoma and it negatively regulated the development of diffuse large B-cell lymphoma and hepatocellular carcinoma, and its lower expression could predict a shorter 5-year survival and overall survival [23, 24]. With regard to GC, compared with the surrounding normal tissues, the expression of TRIM21 in GC tissues was significantly decreased, and the downregulation of TRIM21 expression implied higher recurrence status and lower overall survival [25]. Altogether, these findings suggest a potential tumor-suppressive role of TRIM21. Consistent with their findings, our research reported TRIM21 to be downregulated in GC tissues relative to normal samples. Moreover, patients with low TRIM21 face a poor prognosis. Functionally, we demonstrated by rescue experiments in vitro and in vivo that knocking down miR-99a-3p could reversed cell events induced by TRIM21 downregulation. Acknowledging the observation regarding the observed increase in TRIM21 protein levels compared to baseline (shCtrl) levels, theoretically suggesting that the phenotypic changes should either return to baseline or demonstrate higher levels of apoptosis, less migration, and less proliferation than baseline. However, the lower migration capacity observed only in AGS cells after co-transfection, as opposed to AGS cells in the shCtrl group, presents an interesting discrepancy. Several potential reasons can be considered: Firstly, cell type specificity may contribute, as different cell types exhibit varied responses to the same genetic expression changes due to differences in intracellular signaling pathways, gene regulatory networks, and cellular origins [26]. Additionally, compensatory mechanisms within cells may be activated to counterbalance the effects of increased TRIM21 protein levels [27]. Experimental conditions, while maintaining the same post-transfection measurement time, may introduce variations in drug concentrations or cell culture conditions, influencing cellular responses. Furthermore, the complexity of cellular signaling networks implies that changes in TRIM21 protein levels post co-transfection could trigger intricate signaling cascade alterations, ultimately affecting cellular functions and phenotypes in unforeseen ways.

How does TRIM21 mediate these effects? The potential mechanisms by which TRIM21 exerts its influence on cellular processes are proposed. TRIM21 has been identified as a critical E3 ubiquitin ligase targeting mutant p53 (mutp53) through specific interactions identified in screens for mutp53-interacting proteins. TRIM21 selectively interacts with mutp53, facilitating its ubiquitination and subsequent degradation, thereby mitigating mutp53-mediated tumorigenesis [28]. Given the prevalence of TP53 mutations in human cancers, mutp53 proteins often accumulate to high levels, contributing to cancer progression through gain-of-function (GOF) mechanisms [29]. Notably, gastric cancer patients with TP53 mutations exhibit a more aggressive and chemoresistant phenotype [30]. In light of these findings, we hypothesize that elevated levels of miR-99a-3p may promote gastric cancer progression by inhibiting TRIM21-mediated mutp53 ubiquitination and degradation. However, further experimental validation is required to confirm this proposed mechanism.

In conclusion, this study underscored the importance of the miR-99a-3p/TRIM21 axis in the regulation of tumor growth, thus offering potential insights into therapeutic strategies for gastric cancer.

## Supplementary Information

Below is the link to the electronic supplementary material.

Supplementary file1 (DOCX 15 KB)

## Data Availability

The data used and analyzed during the current study are available from the corresponding author on reasonable request.
