# Supplementary material for: MiR-99a-3p downregulates TRIM21 to promote gastric cancer development
Source: Mol Cell Biochem. 2024 May 8;480(2):1001–12. doi: 10.1007/s11010-024-05005-0 (PMC11835897; doi:10.1007/s11010-024-05005-0)
Supplement: Supplementary file 1 — Supplementary file1 (DOCX 15 KB) [file 11010_2024_5005_MOESM1_ESM.docx]

Table S1. Antibodies used in western blot, RIP and IHC.

| Primary antibodies | Dilution in WB | Source species | Company | Catalog No. |
| --- | --- | --- | --- | --- |
| TRIM21 | 1:1000 | Rabbit | Proteintech | 12108-1-AP |
| N-cadherin | 1:2000 | Rabbit | abcam | ab76011 |
| Vimentin | 1:2000 | Rabbit | abcam | ab92547 |
| Snail | 1:1000 | Rabbit | CST | 3879S |
| Caspase-9 | 1:500 | Rabbit | Abcam | ab52298 |
| Bcl-2 | 1:3000 | Rabbit | Proteintech | 12789-1-AP |
| Bax | 1:2000 | Rabbit | Proteintech | 50599-2-Ig |
| AGO2 | 1:1000 | Mouse | Proteintech | 66720-1-Ig |
| GAPDH | 1:30000 | Mouse | Proteintech | 60004-1-Ig |
|  |  |  |  |  |
| Secondary antibody | Dilution |  | Company | Catalog No. |
| Goat Anti-Rabbit | 1:3000 |  | Beyotime | A0208 |
| Goat Anti- Mouse | 1:3000 |  | Beyotime | A0216 |

| Primary antibodies | Dilution in IHC | Source species | Company | Catalog No. |
| --- | --- | --- | --- | --- |
| TRIM21 | 1:200 | Rabbit | abcam | Ab119895 |
| Ki67 | 1:200 | Rabbit | abcam | ab16667 |
|  |  |  |  |  |
| Secondary antibody | Dilution |  | Company | Catalog No. |
| Goat Anti-Rabbit | 1:400 |  | abcam | A6721 |
